# *Culicoides* and midge-associated arboviruses on cattle farms in Yunnan Province, China[Fn FN1]

**DOI:** 10.1051/parasite/2024072

**Published:** 2024-11-19

**Authors:** Ying-Liang Duan, Zhan-Hong Li, Glenn A. Bellis, Le Li, Bing-Gang Liu, Jian-Ping Wang, Jian-Mei Liu, De-Fang Liao, Jian-Bo Zhu

**Affiliations:** 1 Yunnan Tropical and Subtropical Animal Virus Diseases Laboratory, Yunnan Animal Science and Veterinary Institute Fengyu Road, Panlong District Kunming 650224 Yunnan China; 2 Key Laboratory of Transboundary Animal Diseases Prevention and Control (Co-construction by Ministry and Province), Ministry of Agriculture and Rural Affairs Fengyu Road, Panlong District Kunming 650224 Yunnan China; 3 Research Institute for the Environment and Livelihoods, Charles Darwin University Ellengowan drive Casuarina NT 0810 Australia; 4 Center for Animal Disease Control and Prevention Xinxi Street Lufeng 651200 Yunnan China

**Keywords:** *Culicoides*, bluetongue virus, Yunnan Orbivirus, Yongshan totivirus, Overwinter

## Abstract

*Culicoides* spp. (Diptera: Ceratopogonidae) are small biting midges, some of which are the vectors of arboviruses affecting livestock, *i.e.*, African horse sickness virus (AHSV), bluetongue virus (BTV), and epizootic hemorrhagic disease virus (EHDV). Yunnan Province, located in southwestern China, has a history of high prevalence of arboviruses. The diversity and abundance of *Culicoides* was observed between March 2022 and March 2023 on two cattle farms in Lufeng County of Yunnan Province, China and virus isolation and PCR detection were attempted from cattle blood and *Culicoides* spp. collected from the farms. Light trap collections contained 19 species of *Culicoides* belonging to 8 subgenera and one unplaced species group and were dominated by *C. oxystoma* (63.4%), *C. imicola* (16.2%), *C. arakawae* (13.4%), *C.* sp. near *newsteadi* (2.3%), and *C. orientalis* (1.7%). A total of 8,343 *Culicoides* were used for statistical analysis; from these collections 997 *Culicoides* specimens belonging to 10 species were screened for the presence of BTV, EHDV, Yunnan Orbivirus (YUOV), and Yongshan totivirus (YSToV) using reverse transcription quantitative PCR (RT-qPCR). One strain of YUOV was isolated from cattle blood and 7 isolates of YSToV were isolated from 5 different species of *Culicoides*. BTV and YSToV were detected from 2 and 3 pools of parous *C. imicola* specimens by RT-qPCR, respectively, which is the first report of a totivirus to be associated with *Culicoides*. *Culicoides imicola* is likely to be the major vector of *Culicoides-*borne arboviruses in Lufeng County, which is a relatively dry locality, and adult *C. imicola* may play a role of BTV overwintering.

## Abstract (fr)

Les culicoïdes (Diptera : Ceratopogonidae) sont de petits moucherons piqueurs, dont certains sont les vecteurs d’arbovirus affectant le bétail, à savoir le virus de la peste équine africaine (AHSV), le virus de la fièvre catarrhale (BTV) et le virus de la maladie hémorragique épizootique (EHDV). La province du Yunnan, située dans le sud-ouest de la Chine, a un historique de prévalence élevée d’arbovirus. La diversité et l’abondance des *Culicoides* spp. ont été observées entre mars 2022 et mars 2023 dans deux fermes d’élevage de bovins du comté de Lufeng, dans la province du Yunnan, en Chine, et l’isolement du virus et la détection par PCR ont été tentés à partir du sang de bovins et des *Culicoides* spp. collectés dans les fermes. Les collections de pièges lumineux contenaient 19 espèces de *Culicoides* appartenant à 8 sous-genres et un groupe d’espèces non placé, et étaient dominées par *C. oxystoma* (63,4 %), *C. imicola* (16,2 %), *C. arakawae* (13,4 %), *C.* sp. proche de *newsteadi* (2,3 %) et *C. orientalis* (1,7 %). Un total de 8343 culicoïdes ont été utilisés pour l’analyse statistique; à partir de ces collections, 997 spécimens de *Culicoides* appartenant à 10 espèces ont été examinés pour la présence de BTV, EHDV, Yunnan Orbivirus (YUOV) et Yongshan Totivirus (YSToV) en utilisant la PCR quantitative à transcription inverse (RT-qPCR). Une souche de YUOV a été isolée du sang de bovin et 7 isolats de YSToV ont été isolés de 5 espèces différentes de *Culicoides*. Le BTV et le YSToV ont été détectés respectivement dans 2 et 3 pools de spécimens pares de *C. imicola* par RT-qPCR, ce qui constitue le premier signalement d’un totivirus associé à *Culicoides*. *Culicoides imicola* est probablement le principal vecteur d’arbovirus transmis par culicoïdes dans le comté de Lufeng, qui est un endroit relativement sec, et les adultes de *C. imicola* peuvent jouer un rôle dans l’hivernage du BTV.

## Introduction

*Culicoides* (Diptera: Ceratopogonidae) are small biting insects and at least 40 of the more than 1,300 species of *Culicoides* worldwide are reported as potential vectors for arboviruses [7, 39], including several important livestock viruses such as African horse sickness virus (AHSV), bluetongue virus (BTV), and epizootic hemorrhagic disease virus (EHDV) [36, 42, 49]. Recently, six species, i.e., *Culicoides tainanus* Kieffer, *C.* sp near *obsoletus* Meigen, *C. imicola* Kieffer, *C. jacobsoni* Macfie, *C. orientalis* Macfie, and *C. oxystoma* Kieffer, have been shown to carry arboviruses including BTV, Tibet orbivirus (TIBOV), EHDV, Banna virus (BAV), and Yunnan Orbivirus (YUOV) in Yunnan Province, China [11, 13, 16, 18, 24, 31].

Almost all members of the genus *Orbivirus* of Sedoreoviridae are arboviruses, for example AHSV, BTV, EHDV, Palyam virus, TIBOV, and YUOV [4, 26, 30, 49, 57, 58]. Some of these, like BTV and EHDV, mainly infect ruminants such as cattle, sheep, goats and deer, and are transmitted by *Culicoides* [12, 39, 49].

Totiviridae is composed of a group of viruses with a single dsRNA segment and parasitizing fungi, yeasts, and protozoa [26, 60]. A series of totivirus-like viruses, such as Yongshan totivirus (YSToV), Yuanmou totivirus (YMToV), Tianjin totivirus, and Shanghai totivirus have been reported in China over the past two decades [45, 46, 64]; however, these viruses came from mosquitoes instead of the known hosts of Totiviridae.

In subtropical and temperate zones, summer and autumn are the seasons when BTV is most prevalent among ruminants [35, 48, 63, 67]; however, the virus is often found to be absent during winter yet emerges again in the next warm season [35, 48]. The mechanism of BTV overwintering has been studied by several researchers, but has never been resolved [20, 41, 44, 48, 52, 53, 59].

Lufeng County of Yunnan Province, China is a relatively dry locality and experiences hot summers and cold winters. Quite a few bovine and goats positive for BTV antibodies were reported in three counties (Chuxiong County, Shuangbai County, and Yuanmou County) adjacent to Lufeng County in 2002 [8]. But so far, no disease of bovines, goats or sheep has been publicly reported from Lufeng County. However, five cattle from a farm in Lufeng County had died of disease in the summer in 2021. Subsequently, blood samples of 24 subclinical and healthy cattle were collected in 2021 and submitted to the Yunnan Tropical and Subtropical Animal Virus Diseases Laboratory. Approximately 5 of 24 were positive for BTV RNA, and 4 of 24 were positive for EHDV RNA, through RT-qPCR detection by Le Li *et al*. (unpublished). This event prompted us to investigate the *Culicoides* and the arboviruses, especially BTV and EHDV, on this farm in the next year. Moreover, this is an ideal locality to investigate the overwintering situation of arboviruses in Lufeng County.

This study reports the species diversity and abundance of *Culicoides* and virus isolation and detection from cattle and *Culicoides* on two cattle farms in Lufeng County, Yunnan Province, China.

## Materials and methods

### Ethics approval and consent to participate

The procedure for sampling cattle blood was approved by the Ethics Committee of the Yunnan Animal Science and Veterinary Institute under number YNASVI01-2022LL.

### Collecting samples

**Figure S1:**
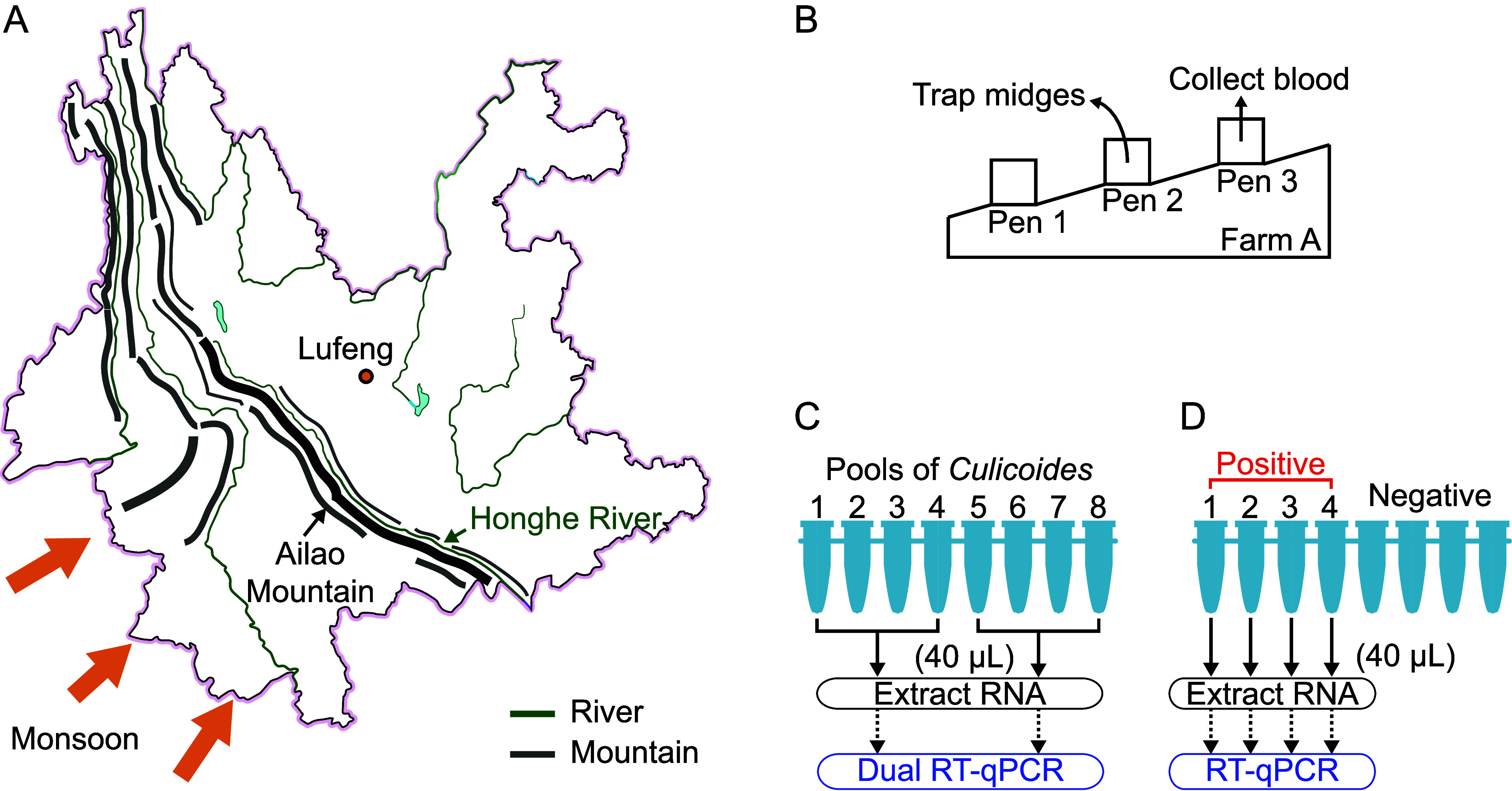
Locality map of Yunnan and flow chart of experiment. (A) Sketch map of Yunnan Province, China. Major rivers and mountains are shown roughly by green and grey lines, respectively. (B) Collecting samples on Farm A. (C) Preliminary RT-qPCR test for *Culicoides*. (D) Further RT-qPCR test for positive samples.

Two cattle farms (farms A and B) in Caiyun town, Lufeng County, Yunnan Province, China were set up as collection sites (Fig. S1A). There is a 3.5 km straight line distance between the two farms. Farms A and B fed approximately 380 cattle in three adjacent pens and 200 cattle in two adjacent pens, respectively (Table S1). As mentioned above, five cattle on farm A had died of disease in 2021. Thereafter, hygiene practices on both farms included twice weekly spraying of pesticides in the pens and regular removal of dung. On each farm, two battery powered UV-light traps (Yaoyu electronics Co. Ltd., Zhangzhou, China) were set; one trap captured living midges into a dry bottle with fresh leaves to provide humidity, while the other collected midges into a bottle containing 75% ethanol (Table S2). The traps ran from approximately 4:00 pm (before sunset) to 9:00 am the following day. Living midges were processed for virus isolation soon after collection, while the midges in ethanol were stored at 4 °C until processed. *Culicoides* were collected on one night each month between March 2022 and March 2023 (Table S2).

Additionally, 39 EDTA^+^ blood samples were collected from 20 cattle on Farm A on 3 occasions between March 4 and June 1, 2022 (Table S2, Fig. S1B), and stored at 4 °C.

### Sorting of *Culicoides*

*Culicoides* collected in ethanol were identified according to the morphological keys of Wirth [61] and Yu *et al*. [66], and graded into blood fed, parous + gravid and nulliparous females, and males. Parous status was identified by the presence of burgundy pigment in the abdomen [19]. Representative specimens of all species were mounted onto glass slides following the methods described by Bellis *et al*. [6]. All specimens collected were counted and the male rate (= male midges/total midges), blood fed rate (= blood fed midges/female midges), and nulliparous rate [= nulliparous midge/(parous midges + nulliparous midges)] were calculated for each collection.

### Weather data

Daily weather data for Lufeng County were collected from the website of Baidu weather [2]. Daily precipitation data in Lufeng County were not available, so precipitation was estimated by the categories of weather. Rainfall over each 24-hour period was classified into five categories: rainstorm (50.0–99.9 mm), heavy rain (25.0–49.9 mm), moderate rain (10.0–24.9 mm), and light rain (0.1–9.9 mm) or overcast by the China Meteorological Administration [1]. No rainstorm or heavy rain was reported in this county during the period we studied. To estimate daily precipitation quantitatively, rainfall scores were designed as 4 points for moderate rain, 2 points for light rain, 0.5 points for overcast or fog, and 0 point for sunny or cloudy.

### Sample preparation for viral isolation

Prior to October 2022, live midges were prepared for viral isolation on the day of collection but thereafter (between October 2022 and March 2023) they were incubated at no less than 12 °C for 24 h before processing.

Midges were immobilized at −20 °C for at least 20 min and parous + gravid female *Culicoides* without a visible blood meal were selected for viral isolation. Conspecific specimens were pooled and placed into 1.5 mL EP tubes with 1 mL sterilized phosphate buffered saline (PBS). Each pool contained between 1 and 35 specimens. PBS was removed following soft shock using a vortex machine for 30 s and centrifugation (1,850 × *g*, 2 min), then the operation was repeated. Subsequently, 600 μL minimum essential medium (MEM) and two sterile steel balls (Φ3 mm) were added to each tube. Specimens were homogenized at 25 Hz for 25 min using a multiple functional homogenizer TissueLyser II (QIAGEN, Hilden, Germany). The homogenates were centrifuged (1,850 × *g*, 2 min), and the supernatants were used for inoculation.

For blood samples, 1 mL blood was centrifuged at 300 × *g* for 5 min, the plasma was removed and the red blood cells were suspended in 1 mL sterile PBS. After centrifugation, PBS was replaced with 1 mL sterile PBS and then the operation was repeated, after which 1 mL of sterile double distilled water was added to suspend the cell pellet, and the suspension was placed on ice for 10 min prior to inoculation onto cell lines.

### Viral isolation

Baby hamster kidney (BHK-21) and *Aedes albopictus* mosquito (C6/36) cell lines were cultured in MEM with 5% fetal bovine serum (FBS), 100 U/mL penicillin and 100 μg/mL streptomycin (Gibco, Thermo Fisher Scientific, Grand Island, NY, USA) at 37 °C and 28 °C, respectively. For viral isolation, BHK-21 and C6/36 cells were prepared individually in 6-well plates and 300 μL of supernatant prepared from midges or blood described above were added to each well. The medium was replaced by 3 mL MEM with 1% FBS at 2 h post inoculation. Infected cells were cultured for 7 days and examined daily for obvious cytopathic effect (CPE). Cells exhibiting CPE were removed to a 5 mL tube and stored at −80 °C. If after 7 days no CPE appeared, the entire plate was frozen and thawed twice, centrifuged at 1,850 × *g* for 3 min and 300 μL of supernatant were inoculated onto new cells and cultured for a further 7 days. Cells were passaged in this manner 3 times then discarded.

### Extraction of nucleic acids from viral isolates

All cell cultures exhibiting CPE were subjected to viral nucleic acid extraction. Briefly, nucleic acids were extracted from 200 μL of cellular medium using a MiniBEST Viral RNA-DNA Extraction Kit (#2158, Takara, Dalian, China), according to the manufacturer’s instructions. Nucleic acids were eluted by 35 μL/sample RNase-free water and kept at −80 °C until use. The samples from the positive cell cultures were used for metagenomic sequencing and viral identification by PCR, respectively.

### Metagenomic sequencing

An aliquot of 10 μL of nucleic acid from up to 8 isolates was pooled and sent to MAGIGENE Science and Technology Ltd for sequencing. Briefly, a DNA library was constructed using an NEB Next^®^ Ultra II™ DNA Library Prep Kit for Illumina (#E7645S, New England Biolabs, Ipswich, MA, USA), according to the manufacturer’s instructions; the quality of the library was checked by Agilent 4200 TapeStation (Agilent Technologies, Santa Clara, CA, USA) using a Qubit^®^ dsDNA HS Assay Kit (Life Technologies, Carlsbad, CA, USA). Samples were sequenced by a Novaseq 6000 System (Illumina, San Diego, CA, USA) using 150 bp paired-end pattern.

### Phylogenetic analysis

Two contigs acquired from metagenomic sequencing, namely contig-214 for the YUOV T2 (VP2) gene and contig-S5 for the YSToV partial genome (Table S3), were used for phylogenetic analysis. Reference sequences of similar viruses were downloaded from NCBI. Sequences were aligned by MUSCLE (Codons) and phylogenetic trees were constructed using a Neighbor-Joining (NJ) algorithm (bootstrap = 1,000), using MEGA 11 software [23]. As some sequences were not complete, the protruding ends of all sequences were truncated following alignment so that they were all of equal length. For T2 gene analysis, the sequences were truncated to begin at the 4th codon and end at the penultimate codon with a length of 2,811 bp for YUOV and middle point orbivirus (MPOV) and 2,694 bp for BTV. For RNA dependent RNA polymerase (RdRP) analysis, sequences were truncated to start at the 1st codon and end at the penultimate codon according to YSToV strain Yunnan/2018, and with a length of 2,031 bp.

### Extracting nucleic acid from midges

Conspecific pools of no more than 5 blood fed female or no more than 10 parous + gravid female midges were tested for the presence of viruses. Each pool of specimens was digested non-destructively in 55 μL tissue lysis buffer (TIANGEN, Tiangen, Beijing, China) containing 0.2 mg/mL proteinase K (TIANGEN) at 25 °C for 16 h [15]. A MagMAX™ Express-24 machine (Ambion, Thermo Fisher Scientific, Waltham, MA, USA) coupled with KingFisher plate (Thermo Fisher Scientific) and MagMAX™-96 Viral RNA Isolation kit (Ambion) were used to extract the nucleic acid from the supernatant, according to the manufacturer’s instructions, and the nucleic acids were eluted in 50 μL/well elution buffer. For preliminary screening, a 10 μL aliquot from 4 pools of insects were pooled and tested (Fig. S1C) and positive pools were investigated further by testing 40 μL of lysate of each of the 4 single pools comprising the positive group (Fig. S1D).

### Reverse transcription and polymerase chain reaction (RT-PCR)

The cDNA of all viral isolates was synthetized by 10 μL of RNA by ProFlex PCR System (ABI, Foster City, CA, USA) using a PrimeScrip 1st Strand cDNA Synthesis Kit and random primers (#6110A, Takara), according to the manufacturer’s instructions.

To identify YUOV and YSToV, 2.5 μL of viral cDNA was added to 22.5 μL of PCR reaction system confected using PrimeSTAR HS (#R040Q, Takara) and primers designed in this study (Table S4), according to the manufacturer’s instructions. The PCR cycling program consisted of: denaturation at 95 °C, 1 min, then denaturation at 95 °C for 10 s, annealing at 55 °C for 5 s, extension at 68 °C for 80 s (YUOV) or 1 min (YSToV) for 30 cycles; and a final extension at 68 °C for 1 min, with storage at 4 °C.

PCR amplified DNA fragments (5 μL per sample) were separated by electrophoresis in 1.5% agarose gel with dye GoldView-II, and screened by a Gel Doc XR^+^ System (Bio-Rad, Hercules, CA, USA).

### Detecting virus in midges by RT-qPCR

A one-step RT-qPCR method was used to scan for 4 viruses (BTV, EHDV, YUOV, and YSToV) in midges. The primers and probes for BTV [25] and EHDV [65] were used from previous reports, while the primers and probes for YUOV and YSToV were novel (Table S4). A 2 μL aliquot of midge sample was added to 20 μL of reaction solution prepared using a Quant One Step PrimeScript RT-PCR Kit (Takara), according to the manufacturer’s instructions. The RT-qPCR was performed on a Fast7500 Realtime PCR machine (ABI) at the following cycling conditions: 42 °C, 5 min; 95 °C, 10 s; 95 °C for 10 s, 60 °C for 34 s, 40 cycles. Fluorescence was measured at the end of each extension step.

For the primary scan, samples were detected by dual channel RT-qPCR for BTV/EHDV and YUOV/YSToV, respectively (Fig. S1C). For further scanning, a candidate sample from a single pool was detected by RT-qPCR for single target in triplicate, according to the result of primary RT-qPCR (Fig. S1D).

### Confirming the BTV in *Culicoides*

To confirm the positive results of RT-qPCR experiments for BTV, primers against BTV NS3 were designed based on the sequence of strain BTV4/YTS-4 (JX560422.1) (Table S4). Detailed methods and results are described in Supplemental File 1.

## Results

### *Culicoides* diversity and abundance

**Figure 1:**
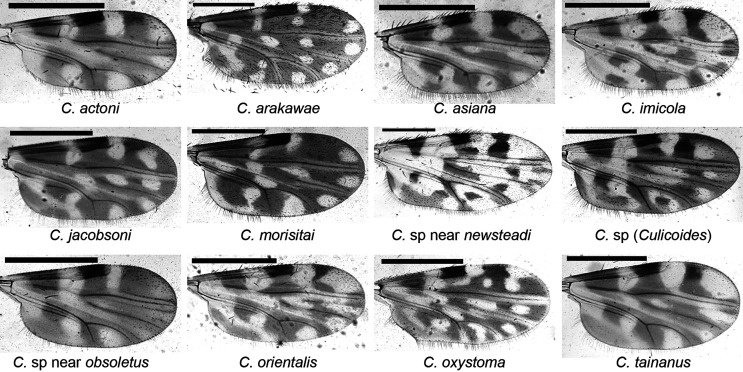
Wings of represented female specimens. The wings of represented *Culicoides* species collected from Lufeng County in this study, scale bar = 0.5 mm.

**Figure 2:**
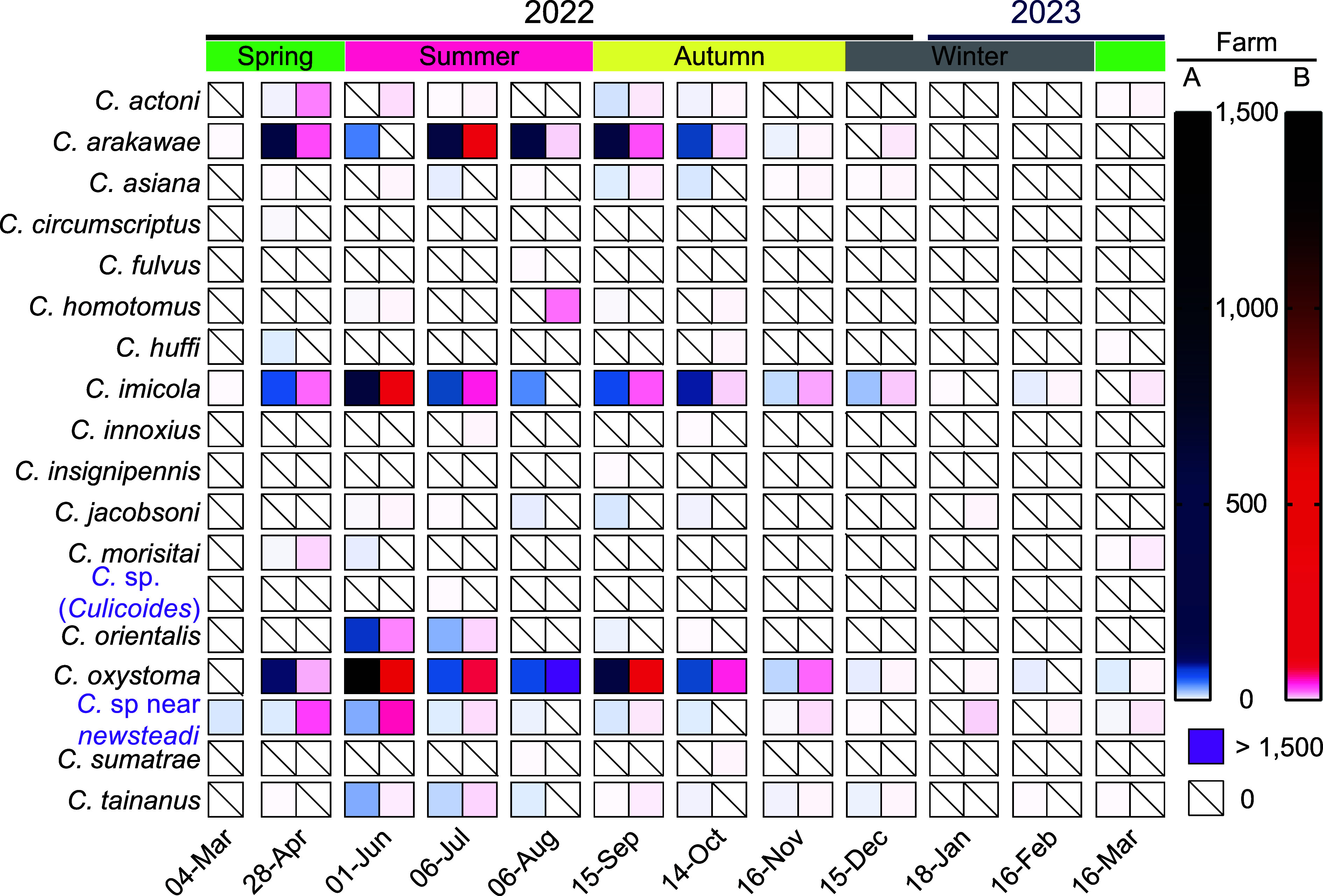
Abundance of *Culicoides* species on two farms in Lufeng Province, Yunnan, China. Each cell represents a collection and the color reflects the *Culicoides* abundance in each collection.

Collections yielded 19 species of *Culicoides* belonging to 8 subgenera (*Avaritia*, *Beltranmyia*, *Culicoides*, *Hoffmania*, *Meijerehelea*, *Monoculicoides*, *Oecacta*, and *Remmia*) and one species group (Clavipalpis group) (Table 1, Fig. 1). A total of 8,343 specimens were collected with UV traps with ethanol and used for statistical analysis, and 754 parous/gravid female specimens without blood meal from living collections were used for viral isolation. Specimens of *C.* sp near *obsoletus*, a species previously reported in Yunnan [13], were only found in the live collections for viral isolation. A novel *Culicoides* species belonging to subgenus *Culicoides* and with similar morphology to *C. neilamensis* Liu & Deng [32] was found. Seasonal abundance of individual species of *Culicoides* on the two farms was in general agreement with summer peaks evident in all species (Fig. 2). According to the number of specimens collected on both farms over the year, the dominant species was *C. oxystoma* (63.4%), followed by *C. imicola* (16.2%), *C. arakawae* Arakawa (13.4%), *C.* sp near *newsteadi* Austen (2.3%; a species reported as *C. punctatus* Latreille by us before) and *C. orientalis* (1.7%), with the remaining species contributing less than 1% (Table 1). Midge abundance was generally quite low with most collections on both farms containing no midges, and all but 5 containing less than 300 specimens (Fig. 2).

**Table 1:** Diversity and abundance of *Culicoides* collected over a 12-month period from 2 farms in Lufeng County, Yunnan Province, China.

Rank^a^	Species	Subgenus	Amount (ratio, %) of *Culicoides* per trap		
			Farm A	Farm B	Average^b^
1	*C. oxystoma*	*Remmia*	1,891 (46.0)	3,402 (80.4)	2,646.5 (63.4)
2	*C. imicola*	*Avaritia*	974 (23.7)	377 (8.9)	675.5 (16.2)
3	*C. arakawae*	*Meijerehelea*	875 (21.3)	244 (5.8)	559.5 (13.4)
4	*C.* sp near *newsteadi*	*Culicoides*	84 (2.0)	107 (2.5)	95.5 (2.3)
5	*C. orientalis*	*Avaritia*	117 (2.8)	22 (0.5)	69.5 (1.7)
6	*C. tainanus*	*Avaritia*	68 (1.7)	11 (0.3)	39.5 (0.9)
7	*C. actoni*	*Avaritia*	21 (0.5)	28 (0.7)	24.5 (0.6)
8	*C. asiana*	*Avaritia*	29 (0.7)	5 (0.1)	17.0 (0.4)
9	*C. homotomus*	*Monoculicoides*	4 (0.1)	23 (0.5)	13.5 (0.3)
10	*C. jacobsoni*	*Avaritia*	23 (0.6)	2 (0.0)	12.5 (0.3)
11	*C. morisitai*	*Oecacta*	10 (0.2)	7 (0.2)	8.5 (0.2)
12	*C. huffi*	Clavipalpis group	9 (0.2)	1 (0.0)	5.0 (0.1)
13	*C. circumscriptus*	*Beltranmyia*	2 (0.0)	0 (0.0)	1.0 (0.0)
13	*C. innoxius*	*Hoffmania*	1 (0.0)	1 (0.0)	1.0 (0.0)
13	*C. sumatrae*	*Hoffmania*	1 (0.0)	1 (0.0)	1.0 (0.0)
14	*C. fulvus*	*Avaritia*	1 (0.0)	0 (0.0)	0.5 (0.0)
14	*C. insignipennis*	*Hoffmania*	1 (0.0)	0 (0.0)	0.5 (0.0)
14	*C.* sp. (*Culicoides*)	*Culicoides*	1 (0.0)	0 (0.0)	0.5 (0.0)
	Total		4112 (100.0)	4231 (100.0)	4171.5 (100.0)

a The rank of species based on the average amounts.

b Average amount = (A amount + B amount)/2, average percentage = average amount/average total amount.

**Figure 3:**
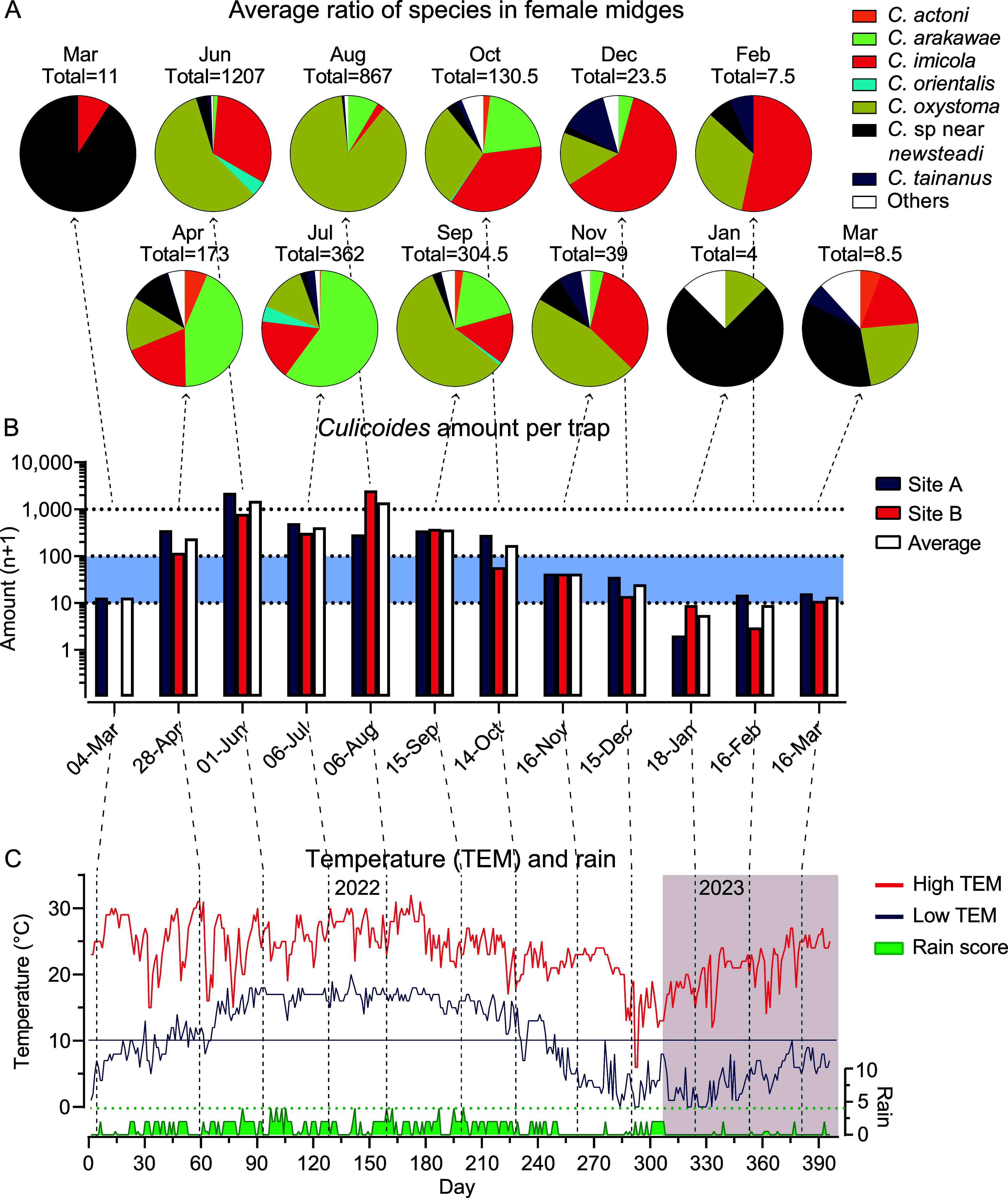
Seasonal abundance of *Culicoides* and weather on 2 farms in Lufeng County, Yunnan, China. (A) The relative proportion of female midges belonging to the major species present in collections from Mar 2022 to Mar 2023. (B) Total midge abundance (log_10_ (n + 1)) on Farm A and Farm B, and the average abundance at the two farms from Mar 2022 to Mar 2023. (C) Daily maximum and minimum temperature (TEM) and rain score between March 1, 2022 and March 31, 2023 in Lufeng County.

Annual weather data between March 1, 2022 and March 31, 2023 in Lufeng showed that daily maximum temperatures ranged from 6 °C to 32 °C, and daily minimum temperatures ranged from 0 °C to 25 °C, while the daily temperature difference ranged from 1 °C to 23 °C. Minimum temperatures changed steadily with the season and rainfall usually lowered the maximum temperature and decreased the average daily difference in temperature (Fig. 3C).

Generally, all species of *Culicoides* were active between late spring and autumn (between April and October) peaking in the summer (Figs. 2 and 3B), showing a correlation with both temperature and rainfall (Figs. 3B and 3C). *Culicoides oxystoma* and *C. arakawae* both showed peak abundance in summer, while *C. imicola* was found in every month, except January 18; *C.* sp. near *newsteadi* became the dominant species in winter (Fig. 3A).

### Proportions of male, blood fed and nulliparous *Culicoides*

Male specimens for 11 species were collected (Table 2). *Culicoides circumscriptus* Kieffer had the highest male: female ratio but only 2 specimens were collected; *C. homotomus* Kieffer and *C. oxystoma* had average male: female ratios above 30%, while the remainder had ratios below 20%.

**Table 2:** Proportion of male specimens in UV trap collections of *Culicoides* collected on 2 farms over 12 months in Lufeng County, Yunnan Province, China.

Species	Male rate		
	Males	Males + females	Proportion (%)
*C. circumscriptus*	2	2	100.0
*C. homotomus*	13	27	48.1
*C. oxystoma*	1,753	5,293	33.1
*C. morisitai*	3	17	17.6
*C. arakawae*	188	1,119	16.8
*C. tainanus*	12	79	15.2
*C. huffi*	1	10	10.0
*C. imicola*	103	1,351	7.6
*C. actoni*	1	49	2.0
*C.* sp near *newsteadi*	2	191	1.0
*C. orientalis*	1	139	0.7

**Figure 4:**
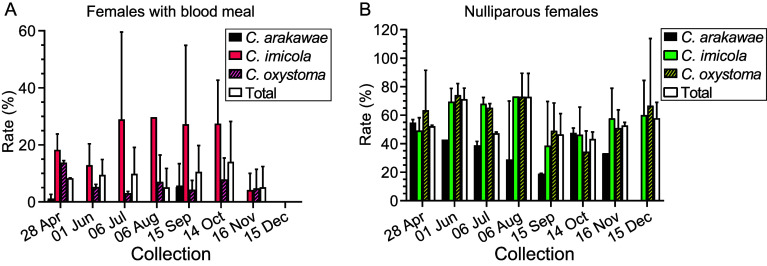
Proportions of blood fed and nulliparous females. The average percentages (mean ± SD) of blood feeding (A) and nulliparous female (B) *Culicoides* collected on 2 farms in Lufeng county, Yunnan, China between April and December 2022.

Seasonal changes in the blood fed and nulliparous rate of *C. arakawae*, *C. imicola*, *C. oxystoma*, and total *Culicoides* between April 28 and December 15 were studied (Fig. 4) as too few specimens of other species were collected to analyze. The blood fed rate of all species ranged from 5.1% to 14.1% between April 28 and November16, while no blood fed *Culicoides* were found in December (Fig. 4A). The proportion of blood fed specimens of *C. arakawae* was lower than that of other species, and was zero in most cases, while the proportion of blood fed *C. imicola* was higher than other species, often around 30% (Fig. 4A). The nulliparous rates of *Culicoides* were not significantly correlated with the season; generally, the nulliparous rate of *C. arakawae* was lower than that of other species, while the values of *C. imicola* and *C. oxystoma* were synchronous with the total *Culicoides* (Fig. 4B).

### Viral isolates

**Figure S2:**
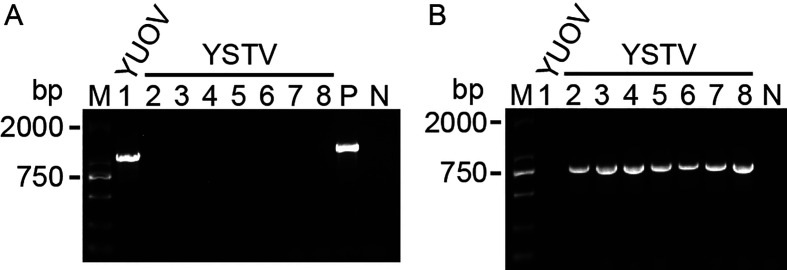
Identification of viral isolates by RT-PCR.

Virus isolation was attempted on 39 blood samples from 20 cattle and 754 specimens of *Culicoides* in 95 pools (Table S5), and in total, 8 viruses were isolated. These isolates were acquired from C6/36 culture and none produced CPE in BHK-21 cells. Metagenomic sequencing produced 11 confirmed contigs from YUOV and YSToV (Table S3), which cued that the unknown viral isolates belonged to these two viruses. Subsequently, the isolates were identified by specific RT-PCR (Fig. S2) and confirmed by sequencing using PCR amplified fragments (Table S3). As a result, a single isolate of YUOV was obtained from cattle and 7 isolates of YSToV were isolated from 7 pools of conspecific midges representing 5 species, i.e., *C. arakawae*, *C. asiana* Bellis, *C. orientalis*, *C. oxystoma*, and *C. tainanus* (Table 3). The 7 YSToV isolates belonged to a single strain based on the sequences of PCR fragments.

**Table 3:** Viruses isolated from pools of *Culicoides* and cattle blood collected from 2 farms in Lufeng County, Yunnan Province, China.

Virus	Isolate voucher	Host	Farm	Number of midges
YUOV	LF6-4	Cattle	A	–
YSToV	LF6C1	*C. orientalis*	A	2
	LF6C2	*C. asiana*	A	1
	LF6C4	*C. tainanus*	A	2
	LF6C5	*C. arakawae*	A	35
	LF6C6	*C. oxystoma*	A	2
	LF6C7	*C. arakawae*	B	1
	LF6C12	*C. arakawae*	B	35

Note: above samples were all collected on July 6, 2022.

**Figure 5:**
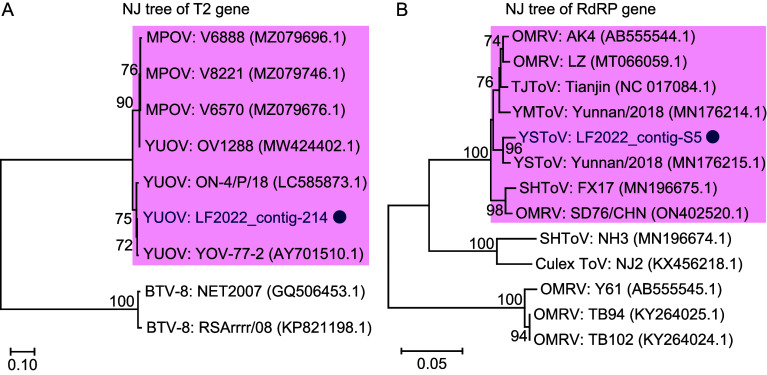
Phylogenetic analysis for YUOV and YSToV, and related viruses. Phylogenetic trees of T2 genes of YUOV, MPOV, and BTV (A), and RdRP genes of YSToV and close totivirus (B) were constructed by NJ algorithm. The sequences of our isolates (contig-214, contig-S5) were marked by circles. Bootstrap values less than 70% were omitted.

In the phylogenetic analysis, the YUOV isolate (contig-214) grouped with a cluster of other YUOV and MPOV viruses (Fig. 5A). The YSToV LF2022 (contig-S5) grouped closely with one other strain of YSToV but also with strains of Yuanmou totivirus (YMToV), Tianjin totivirus (TJToV), Shanghai totivirus (SHToV), and 3 strains of Omono River virus (OMRV) (Fig. 5B).

### RT-qPCR scanning for viruses in midges

A total of 997 specimens placed into 144 pools of conspecific midges representing 10 species were tested by RT-qPCR for the presence of BTV, EHDV, YSToV, and YUOV (Table S6). Two pools of parous *C. imicola* were positive for BTV although the Ct values were high (31.5 and 36.4, respectively) while 3 pools of parous *C. imicola* were positive for YSToV (Table 4). The positive results of BTV in *C. imicola* were confirmed by amplification and sequencing of the BTV NS3 fragments (Supplemental File 1). The midges collected on November 16 had obviously lower BTV abundance (Table 4), and the positive rate of BTV among *C. imicola* was approximately 0.6% (2/331) (Table S6).

**Table 4:** RT-qPCR results from pools of parous + gravid *Culicoides* collected in Lufeng County, Yunnan Province, China.

Pool	Collection code	Collection date	Experiment number	Specimens number in pool	Virus	Ct value^a^
*C. imicola*	P3B	Jun 1, 2022	A46	10	BTV	31.5 ± 0.3
	P8A	Nov 16, 2022	B3	8	BTV	36.4 ± 0.3
	P3B	Jun 1, 2022	A46	10	YSToV	35.9 ± 0.3
		Jun 1, 2022	A47	10	YSToV	36.8 ± 0.2
		Jun 1, 2022	A48	10	YSToV	35.1 ± 0.4

a Ct value is shown as mean ± SD calculated from three replicates of reactions.

## Discussion

Lufeng County, located in the central part of Yunnan, experiences a relatively dry climate with warm days (in summer and autumn) and cold nights. The Ailao Mountain range prevents the northward movement of the summer monsoon from the Indian Ocean, so north of this range, the climate is relatively dry. However, cattle dying of disease on a farm (Farm A) in Lufeng County in 2021 prompted us to begin an investigation of *Culicoides* and midge-associated arboviruses in the following year.

More than 9,000 specimens of *Culicoides* representing 19 species were collected from two cattle farms over a 12-month period. It is possible that the hot, dry days in summer and autumn, coupled with cold nights during winter, and regular spraying of pesticides on dung may have contributed to the low abundance of midges on the two farms. The dominance of *C. oxystoma*, *C. imicola*, and *C. arakawae* has also been observed on livestock farms in some parts of southern Asia [3, 10, 14, 16, 22]. However, this county has distinctive diversity of *Culicoides* compared to other counties in Yunnan.

Notably absent from collections in this study were species belonging to *C.* subgenus *Trithecoides*, as these species are common and often dominant on livestock farms in south Yunnan and other places in southern Asia [16, 22, 61]. Similarly, only 5 specimens of subgenus *Hoffmania* were collected; however, this is consistent with previous reports from Yunnan that *C.* (*Hoffmania*) species prefer altitudes below 11,00 m [15], while these collection sites are approximately 1,400 m above sea level. Previous studies have reported that *C. tainanus* is widespread and common in Yunnan, while *C. imicola* is relatively rare [13, 14, 16, 33]. At Lufeng, however, *C. imicola* are obviously more abundant than *C. tainanus*, although the reasons for this are unclear. This study provides only the second record of *C. morisitai* Tokunaga from Yunnan [33, 66]. It is interesting that *C. morisitai* was absent in summer and autumn in this investigation, while the researchers always collected *Culicoides* in these seasons, so this species might be missed.

Low numbers of specimens of a species close to *C. obsoletus* were collected but only in the live collections. DNA barcodes of this species differ significantly from European populations of *C. obsoletus* [13], suggesting that these represent two distinct species, despite their morphological similarity. *Culicoides obsoletus* has been reported from various places in Yunnan [10, 13, 33]. However, these reports are all based on morphological identification and need to be checked to ensure they do not belong to this cryptic species. Female specimens of several species in the Obsoletus complex are morphologically indistinguishable [38] so examination of male specimens will be needed to clarify the status of these species.

A single specimen found in this study appears to belong to an undescribed species belonging to *C.* subg. *Culicoides*. This new species belongs to a complex of species similar to *C. nielamensis* that we have reported from Shangri-La [13] and found at other high altitude sites in Yunnan. None of these species have the poststigmatic pale spot in cell r_3_ reaching vein M1 which is apparently a characteristic of *C. nielamensis* (Fig. 1) [13, 32]. Further study, including examination of reference specimens of *C. nielamensis* is needed to clarify the status of these species.

*Culicoides punctatus* and *C. newsteadi* identified in Europe were similar in morphology [5], and a similar species complex was reported in Asia [14, 27, 28]. A re-examination of the specimens previously reported as *C. punctatus* by Matsumoto *et al*. [37], Kim *et al*. [27, 28], and Duan *et al*. [14] and probably other studies in Asia, has prompted us to place these specimens closer to *C. newsteadi* than to *C. punctatus* in morphology (Fig. 1), although DNA barcode data indicate that Asian populations are actually different to both of these species [37] (Duan *et al*. unpublished data), so we prefer to refer to this species as *C.* sp. near *newsteadi*.

Three proven, *C. actoni*, *C. fulvus* and *C. imicola* [36, 47, 51], and several suspected vectors of BTV, which were shown to carry BTV in the field, *C. circumscriptus*, *C. jacobsoni*, *C.* sp near *newsteadi*, *C. oxystoma*, *C.* sp near *obsoletus*, and *C. tainanus* [13, 16, 21, 31] were collected at Lufeng, although the relative importance of the species in BTV epidemiology is difficult to assess. Only *C. imicola* was found to be carrying BTV in this study. Additionally, only low numbers of *C. actoni*, *C. jacobsoni*, *C.* sp. near *newsteadi*, *C.* sp near *obsoletus*, and *C. tainanus* and none of *C. fulvus* were tested so it is difficult to assess these species.

Compared to some studies, the Ct values (31.5 and 36.4) for BTV were relatively high in this study, which might be caused by a less optimal method of collecting midges, since systemic errors could be caused by the quality of specimen preservation, efficiency of nucleic acid extraction, or reagents for molecular experiment. In this study, midges for sorting were directly collected in a bottle loading 75% ethanol and connecting to the UV-trap. However, the ethanol began to volatilize as soon as the trap was set. So the trapped midges would soak in low concentrations of ethanol for hours before collecting. Virions in midges might be digested by intracellular proteinases and nucleases partly during the process of apoptosis or necrosis of midge cells. Therefore, the Ct values for BTV in *Culicoides* specimens might be underestimated in this study. Low Ct values (between 20 and 25) for BTV could be acquired in the experiments of artificial infection on *Culicoides* in laboratory [54, 56]. For field midges, collecting midges using a mesh bag under the UV-trap might be better to preserve the midges and viruses [13, 16]. While high Ct values (between 30 and 50) for BTV were acquired from some investigation for field collected midges [21, 31].

The life cycle of *Culicoides* undergoes 4 stages, namely egg, larva, pupa, and adult [48]. Adult female midges typically mate, take a blood meal, lay eggs, and then take another blood meal and produce eggs in a continuous cycle. Meantime, sucking blood is the critical stage to spread arbovirus among hosts of *Culicoides*. Therefore, blood feeding is the index of both midge reproduction and viral transmission, while nulliparous midges represent newborn adults. As male midges never suck blood, they will in theory appear near their habitat or plant food. The relatively high proportion of male specimens of *C. imicola* and *C. tainanus* was unexpected as they are usually uncommon in most collections in Yunnan [13, 16] and in South Korea [28].

*Culicoides arakawae* is considered to be a bird feeder [29]. Relatively high male: female ratios of this species compared to other species have been reported in light traps [28], suggesting either a greater attraction of males of this species to lights or cattle compared to males of other species. The very low number of blood fed female *C. arakawae* collected in Lufeng is likely due to their preference to feed on birds [29], although the possibility that blood fed females are not attracted to lights cannot be ruled out. The high abundance of this species is consistent with other studies [16, 28] and we assume this species is highly attracted to lights, even if traps are not set close to hosts. In this study, *C. imicola* had a higher proportion of blood fed females than other species, and although this does not necessarily equate to a high attack rate on cattle, it does suggest that this species is feeding on the cattle at this site and, given its proven status as a vector of BTV, likely involved in the transmission of virus.

BTV and EHDV are important livestock arboviruses [35, 43, 49, 62] that are prevalent among domestic ruminants in Yunnan Province but have not been reported to cause clinical disease [12, 17, 31] since the first bluetongue cases of 430 sheep in mainland China (Shizong County of Yunnan Province) in 1979 [67]. In summer 2021, 5 cattle died of disease on Farm A in Lufeng County of Yunnan, China. Subsequently, a few blood samples collected from the cattle on the same farm were positive for BTV, EHDV, or for both viruses (Li *et al*., unpublished). Therefore, the farm owner enhanced cleaning on the two farms (sites A and B). In this study, two pools of *C. imicola* from June 1, 2022 and November 16, 2022, respectively were positive for BTV. The absence of BTV in cattle blood and in blood fed midges suggests a low prevalence of BTV on these farms between March 2022 and March 2023. It seems that hygiene practices effectively reduced the prevalence of BTV and EHDV on this farm. The higher Ct value in the positive midge collected in November is consistent with the theory that the RNA polymerase of BTV would be inhibited below 10 °C [55].

BTV has to subsist on its hosts of ruminants and *Culicoides*. However, ruminants such as cattle will eliminate the invaded BTV by specific immunity within months since first infection [50], or killed by the disease in a short time. The *Culicoides* are short-lived, whether the BTV persistently infects single *Culicoides* or not. Therefore, BTV should continuously change host between ruminant and *Culicoides*. In subtropical and temperate zones, BTV prevalence among ruminants in summer and autumn is synchronized with the activity of *Culicoides* [35, 48]. However, cases of BTV decline as the abundance of *Culicoides* declines in winter. So far, the mechanism of BTV overwintering in temperate climates is uncertain [20, 48, 53]. As BTV does not have a DNA genome, it should not have latent infection patterns in ruminants, theoretically and empirically. But it was reported that BTV can be alive in sheep skin for 9 weeks, which is longer than the time of viremia [53]. There is no evidence of vertical transmission of BTV in *Culicoides* [44]. But BTV might overwinter through adult *Culicoides* [20, 52]. In this study, low levels of BTV RNA were detected from a pool of *C. imicola* in November, which partly supports the mechanism of BTV overwintering through adult *Culicoides*. Low levels of BTV in adult *Culicoides* are a balance of coexistence between BTV and *Culicoides*. Because lower abundance of virions means less harm for the host, this theoretically delayed the death of *Culicoides* caused by infection. Furthermore, adult *Culicoides* live longer at lower temperatures [34]. Although this investigation suggested that *C.* sp near *newsteadi* could endure lower temperatures compared with other species, according to increasing ratios of *C.* sp near *newsteadi* in winter; no BTV was detected in specimens from this species, which might be caused by a relatively lower amount.

YUOV was first identified in Yunnan, China [4] and, based on limited field research, was considered to be a mosquito-borne arbovirus [4, 40, 57]. The negative YUOV isolation and PCR results from *Culicoides* coupled with repeated isolation of virus from cattle blood suggest that cattle are a vertebrate host, but *Culicoides* are not important vectors of this virus. The phylogenetic analysis suggested that YUOV and MPOV found in Australia [9] were the same virus species.

The isolation of YSToV from 5 species of *Culicoides* was surprising as Totiviruses have so far only been associated with fungi, yeasts, and protozoa [26, 45, 46], so the presence of a totivirus in insects was surprising. It is possible that the YSToV isolates came from fungi present either internally or externally on the midges as reported for bats by Yang *et al*. [64]. However, YSToV was isolated in C6/36 mosquito cells in this study, which illustrates that YSToV can proliferate in insect cells. Additionally, some totiviruses have been isolated and detected frequently in mosquitos, suggesting more than a passing association with these insects [45, 46]. No vertebrate hosts have been found for these viruses so it is not clear if mosquitoes or midges are acting as vectors or are merely hosts of YSToV. The phylogenetic analysis suggested that YSToV was very closely related to some but not all strains of YMToV, TJToV, SHToV, and OMRV, suggesting that the relationships between these viruses are more complex than currently thought, and more work is required to clarify the status of these viruses.

All the YSToV isolates were acquired from the midges collected on July 6, 2022, suggesting a very high circulation of YSToV among midges in July. The cause is uncertain according to existing information. As the *C. arakawae* reached a very high proportion (>50%) on July 6, 2022 (Fig. 3A), *C. arakawae* might be a major host of YSToV, therefore increasing the circulation of YSToV among *Culicoides* in July. It is unknown whether fungi, yeasts, or protozoa are hosts of YSToV. Presumably high temperature and humidity would promote the growth of these hosts and increase the amount of virus in the environment and expose more midges to infection. However, the air humidity at the collection sites changes significantly during the day, so it is not possible to measure and compare the humidity changes month by month.

## Conclusions

In total, 19 species of *Culicoides* belonging to 8 subgenera and one species group were found in Lufeng County, Yunnan, China between March 2022 and March 2023. The dominant species was *C. oxystoma*, followed by *C. imicola*, *C. arakawae*, *C.* sp near *newsteadi*, and *C. orientalis*. Three well-known BTV vectors (*C. actoni*, *C. fulvus*, and *C. imicola*) and 6 potential BTV vectors (*C. circumscriptus*, *C. jacobsoni*, *C.* sp near *newsteadi*, *C. oxystoma*, *C. tainanus*, and the *C.* sp near *obsoletus*) were collected. However, *C. imicola* was considered the major vector of *Culicoides-*borne arboviruses in this county, which is a relatively dry area with hot summers and cold winters. Furthermore, adult *C. imicola* may play an important role in BTV overwintering here. *Culicoides arakawae* is considered not to be a cattle feeder, although they can sometimes be abundant on cattle farms. One strain YSToV containing 7 viral isolates was the first totivirus to be isolated from *Culicoides*, but more work is required to clarify the status of these viruses, their hosts, and their ability to cause disease.

## Data Availability

Data supporting the conclusions of this article are included within the article and its additional file. The datasets used and/or analyzed during the present study are available from the corresponding author upon reasonable request.

## Supplementary material

The supplementary material for this article can be found at https://www.parasite-journal.org/10.1051/parasite/2024072/olm.

*Table S1*: Details of collection sites in Lufeng County, Yunnan Province, China. *Table S2*: Blood samples and insect collections collected in this study. *Table S3*: High quality contigs and PCR fragments acquired in this study. *Table S4*: Novel primers and probes designed in this study. *Table S5*: Details of the pools of *Culicoides* used in virus isolation attempts. *Table S6*: Species composition and size of pools of *Culicoides* processed for the presence of virus by RT-qPCR. *Supplemental File 1*: Methods and results to confirm BTV in *Culicoides*.
