# Supplementary material for: Culicoides and midge-associated arboviruses on cattle farms in Yunnan Province, China
Source: Parasite. 2024 Nov 19;31:72. doi: 10.1051/parasite/2024072 (PMC11578047; doi:10.1051/parasite/2024072)
Supplement: Supplementary file 1 — Details of collection sites in Lufeng County, Yunnan Province, China. [file parasite-31-72-s1.pdf]

Table S1. Details of collection sites in Lufeng County, Yunnan Province, China.

Farm	Number of lairs	Total number of cattle	Exact coordinates			Straight stretch (m)
			Latitude (°N)	Longitude (°E)	Elevation (m a.s.l.)	
A	3	380	25.00	101.91	1,414	3,485 [a)]
B	2	200	25.03	101.90	1,386	

a) The straight distance between sites A and B was calculated by Vincenty's formulae, according to the latitudes and longitudes of the two sites.
