# Supplementary material for: Culicoides and midge-associated arboviruses on cattle farms in Yunnan Province, China
Source: Parasite. 2024 Nov 19;31:72. doi: 10.1051/parasite/2024072 (PMC11578047; doi:10.1051/parasite/2024072)
Supplement: Supplementary file 2 — Blood samples and insect collections collected in this study. [file parasite-31-72-s2.pdf]

Table S2. Blood samples and insect collections collected in this study.

Lot	Date	Farm A			Farm B	
		Blood (plasma)	Live collection	Ethanol-preserved collection	Live collection	Ethanol-preserved collection
P1	Mar 4, 2022	20	2	2	NA	NA
P2	Apr 28, 2022	10	1	1	NA	1
P3	Jun 1, 2022	9	1	1	1	1
P4	Jul 6, 2022	NA	1	1	1	1
P5	Aug 6, 2022	NA	1	1	1	1
P6	Sep 15, 2022	NA	1	1	1	1
P7	Oct 14, 2022	NA	1	1	1	1
P8	Nov 16, 2022	NA	1	1	1	1
P9	Dec 15, 2022	NA	1	1	1	1
P10	Jan 18, 2023	NA	1	1	1	1
P11	Feb 16, 2023	NA	1	1	1	1
P12	Mar 16, 2023	NA	1	1	1	1

NA: not available.
