# Supplementary material for: Culicoides and midge-associated arboviruses on cattle farms in Yunnan Province, China
Source: Parasite. 2024 Nov 19;31:72. doi: 10.1051/parasite/2024072 (PMC11578047; doi:10.1051/parasite/2024072)
Supplement: Supplementary file 3 — High quality contigs and PCR fragments acquired in this study. [file parasite-31-72-s3.pdf]

Table S3. High quality contigs and PCR fragments acquired in this study.

Virus	Segment/gene	Voucher number	Length (bp)	Completeness (%)	NCBI accession	Best match on NCBI	
						Accession	Identity (%)
YUOV	Seg1	Contig-114	3969	98.80	PP782322	LC585872.1	97.26
	Seg2	Contig-214	2877	99.00	PP782323	NC_007657.1	99.27
	Seg3	Contig-54	2665	99.14	PP782324	NC_007658.1	99.14
	Seg4	Contig-374	1970	98.85	PP782325	NC_007659.1	99.24
	Seg5	Contig-454	1937	98.72	PP782326	NC_007660.1	98.14
	Seg6	Contig-73	1663	98.81	PP782327	NC_007661.1	98.50
	Seg7	Contig-84	1476	98.01	PP782328	LC585878.1	98.78
	Seg8	Contig-34	1165	97.82	PP782329	NC_007663.1	98.97
	Seg9	Contig-313	1063	98.24	PP782330	NC_007664.1	97.74
	Seg10	Contig-37	816	98.91	PP782331	NC_007665.1	99.02
YSToV	Genome	Contig-S5	7357	96.66	PP782332	MN176215.1	96.72
YUOV	VP2	NA	1009	NA	PP782333	NC_007657.1	99.11
YSToV	RdRP	NA	681	NA	PP782334	MN176215.1	98.09
