# Supplementary material for: Culicoides and midge-associated arboviruses on cattle farms in Yunnan Province, China
Source: Parasite. 2024 Nov 19;31:72. doi: 10.1051/parasite/2024072 (PMC11578047; doi:10.1051/parasite/2024072)
Supplement: Supplementary file 4 — Novel primers and probes designed in this study. [file parasite-31-72-s4.pdf]

Table S4. Novel primers and probes designed in this study.

Application	Target virus and gene	Primer/probe	Sequence (5' to 3')	Product length (bp)
PCR	YUOV (T2)	YUOV-F	ATTTACATATCTCCTCCCGCAAG	1,122
		YUOV-R	ATCCGAATTCACCCAAATAGCAA	
	YSToV (RdRP)	YSToV-F	GACTAATCGCGCTATTCCTTC	760
		YSToV-R	TGCCACACGCTATGTTGATT	
	BTV (NS3)	BTV-S10-F	GTTAAAAAGTGTCGCTGCCA	805
		BTV-S10-R	CGCACCCTCCCCCGTTATA	
RT-qPCR	YUOV (T2)	YUOV-qF	GCGTTACAGGAAATTCTTG	77
		YUOV-qR	GCAGTTTCAATCTCTTGTC	
		YUOV-P	FAM-CTGACCACGCAACTGAGACTCT-BHQ1	
	YSToV (capsid)	YSToV-qF	GTTCCGGCGACGAAGGCA	126
		YSToV-qR	AGAGCTGGAAGTAGGTGGACTC	
		YSToV-P	VIC-CAAGCTTGCCGAGTACTTCAGATCC-BHQ1	
