# Supplementary material for: Culicoides and midge-associated arboviruses on cattle farms in Yunnan Province, China
Source: Parasite. 2024 Nov 19;31:72. doi: 10.1051/parasite/2024072 (PMC11578047; doi:10.1051/parasite/2024072)
Supplement: Supplementary file 5 — Details of the pools of Culicoides used in virus isolation attempts. [file parasite-31-72-s5.pdf]

Table S5. Details of the pools of *Culicoides* used in virus isolation attempts.

Collection date	Laboratory incubation [a]	Farm A		Farm B	
		Pool [b]	*Culicoides* [c]	Pool [b]	*Culicoides* [c]
Apr 28, 2022	No	0/8	123 (7)	0/0	0 (0)
Jun 1, 2022	No	0/8	111 (7)	0/7	85 (7)
Jul 6, 2022	No	5/6	51 (6)	2/6	54 (5)
Aug 6, 2022	No	0/7	55 (6)	0/8	54 (6)
Sep 15, 2022	No	0/5	45 (5)	0/7	46 (4)
Oct 14, 2022	Yes	0/8	48 (8)	0/3	25 (3)
Nov 16, 2022	Yes	0/5	18 (5)	0/5	13 (5)
Dec 15, 2022	Yes	0/4	11 (4)	0/2	7 (2)
Jan 18, 2023	Yes	0/0	0 (0)	0/3	3 (3)
Feb 16, 2023	Yes	0/2	3 (2)	0/0	0 (0)
Mar 16, 2023	Yes	0/1	2 (1)	0/0	0 (0)
Total		5/54	467 (13)	2/41	287 (10)

a) Collections were cultured in the laboratory for at least 24 h before isolation attempts.

b) The number of pools is shown as positive/total.

c) *Culicoides* used for isolation and shown as “specimen number (species number)”
