# Supplementary material for: Culicoides and midge-associated arboviruses on cattle farms in Yunnan Province, China
Source: Parasite. 2024 Nov 19;31:72. doi: 10.1051/parasite/2024072 (PMC11578047; doi:10.1051/parasite/2024072)
Supplement: Supplementary file 6 — Species composition and size of pools of Culicoides processed for the presence of virus by RT-qPCR. [file parasite-31-72-s6.pdf]

Table S6. Species composition and size of pools of *Culicoides* processed for the presence of virus by RT-qPCR.

Pool		Month of collection												Total number	
Species	Status	Mar	Apr	Jun	Jul	Aug	Sep	Oct	Nov	Dec	Jan	Feb	Mar	Pool	specimen
C. actoni	p		2	1			1	1	2					7	30
C. arakawae	p		5	1	9	3	4	1	1	1				25	232
C. asiana	bf				1			1						2	8
	p						1							1	3
C. huffi	p		1	1										2	6
C. imicola	bf		2	4	7									13	63
	p		3	13*	2	1	3	4	2*	2		1	1	32	268
C. jacobsoni	p						1							1	4
C. sp near *newsteadi*	bf			3	1		1	1						6	30
	p	1	3	2			2				1		2	11	44
C. orientalis	bf			2	2									4	18
	p			2	1									3	23
C. oxystoma	bf				1				1					2	4
	p		2	7	2	3	6	2	2	1		1	1	27	239
C. tainanus	bf				2							1		3	10
	p			1	1	1			1	1				5	15
Total		1	18	37	29	8	19	10	9	5	1	3	4	144	997

* indicates pools that were positive for BTV in the qPCR test.
