# Supplementary material for: Culicoides and midge-associated arboviruses on cattle farms in Yunnan Province, China
Source: Parasite. 2024 Nov 19;31:72. doi: 10.1051/parasite/2024072 (PMC11578047; doi:10.1051/parasite/2024072)
Supplement: Supplementary file 7 — Methods and results to confirm BTV in Culicoides. [file parasite-31-72-s7.pdf]

Appendix 1 Methods and results to confirm BTV in *Culicoides*

1) One step RT-PCR

One step RT-qPCR method exclusive probe was used to amplify the BTV NS3 fragments from the two RNA samples of *Culicoides* (A46 and B3) positive for BTV. The reaction solution (25 μl) was confected using the Quant One Step PrimeScript RT-PCR Kit (Takara) according to the manufacturer's instructions, containing 2.5 μl of RNA and 20 μM of specific primers. The RT-PCR cycling program consisted of: 50°C, 10 min; 94°C, 2 min; 94°C for 30 s, 55°C for 30 s, 72°C for 1 min, 33 cycles (for A46) or 37 cycles (for B3).

2) Sequencing

The two PCR products were sent to Kunming Shuoqing Biological Technology Company (China) for Sanger sequencing with an ABI3739XL machine (Applied Biosystems). The overlap regions of the forward sequences and reverse sequences were employed.

3) Sequences

>A46 BTV-NS3

ACCTCGGGGCGCCACTCTACCTACTGATCTTAGGTTAATGGTAGTTCGAAACCATCTAG
CGGAACTGACGAGAACTCAGTGAAACTCATCCTCACCGCATCATTGTATGATTGCTTCT
TCATAACTTCCTTCTTGATCATGTCAATCTGCTGATTTAACCCTCTTTCGCTCTTCGCGC
AAACCATCATTATGAAAGTCGCACCTAGATTTACCACTCCGAGCATTGGATTTAGACTC
TTAAACCACTGTGGTATTTCCGCTGATGTACCATTAAGTTTAAAAGCCACACTCATATCA
CTTGAAAGCGTGCATACTGACGTCAGCAATGCTACGACAGCGGCAACTAACAACGTCA
TGTGTATGATAGCTCTCTTTTTCTTTAAACCACCTAGATCACTCTTTAACTTTGGGAGAA
TCTGTTCATTAACATGACGTTTGATCTGTCTTAGCCTCACATCATCACGAAACGCTTCTG
CGTACGATGCGAATGCAGCTTTCTCCGCCTTCTGTGTTTGCGTTGCACCAGTTGTGTTT
GACATCGCTTTATCCAATATTTCAAGGGCAACGGTTGGCATTGATGATGGCATTGGGGC
ACTCGGAGCATATCTCGGTGGTTGCGAAATTGTATCATCCACACGAACCAAACTTAGCT
CTTCGACTCTCTCCTGATTATGTTTCATTTTTTCTTCCTCGAACCTTTGGATCAGCCCGG
ATAGCATGGCAGCG

>B3 BTV-NS3

AAGAGCTAAGTTTGGTTCGTGTGGATGATACAATTTCGCAACCACCGAGATATGCTCCG
AGTGCCCCAATGCCATCATCAATGCCAACCGTTGCCCTTGAAATATTGGATAAAGCGAT
GTCAAACACAACTGGTGCAACGCAAACACAGAAGGCGGAGAAAGCTGCATTCGCATC
GTACGCAGAAGCGTTTCGTGATGATGTGAGGCTAAGACAGATCAAACGTCATGTTAAT

GAACAGATTCTCCCAAAGTTAAAGAGTGATCTAGGTGGTTTAAAGAAAAAGAGAGCTA
TCATACACATGACGTTGTTAGTTGCCGCTGTCGTAGCATTGCTGACGTCAGTATGCACG
CTTTCAAGTGATATGAGTGTGGCTTTTAAACTTAATGGTACATCAGCGGAAATACCACA
GTGGTTTAAGAGTCTAAATCCAATGCTCGGAGTGGTAAATCTAGGTGCGACTTTCATAA
TGATGGTTTGCGCGAAGAGCGAAAGAGGGTTAAATCAGCAGATTGACATGATCAAGAA
GGAAGTTATGAAGAAGCAATCATACAATGATGCGGTGAGGATGAGTTTCACTGAGTTC
TCGTCAGTTCCGCTAGATGGTTTCGAACTACCATTAACCTAAGATCAGTAGGTAGAGTG
GCGCCCCGAGGT
